# Supplementary material for: Transcriptomic Analysis Reveals the Opposite Regulatory Effects of WRKY and CAMTA Transcription Factors on Total Tannin Production in Quercus fabri Fruit
Source: Int J Mol Sci. 2024 Dec 6;25(23):13103. doi: 10.3390/ijms252313103 (PMC11642043; doi:10.3390/ijms252313103)
Supplement: Supplementary file 1 [file ijms-25-13103-s001.zip › Supplementary Document S1.pdf]

Supplementary Document S1. CDS (coding sequence) of WRKY and CAMTA

LOC115955609 (WRKY):

ATGGCCTCCACTTCATGTTTCTTGCACCACCATGCACTTACCACTCCCACTAGAGCCTC
ATCATCACAGCGCCAAGTGCCAAACATGAAGCCCACTCAGGTAGTCTGCAAGGCACA
GAAGCAGTCAGTTCATGAAGATGATATGAGTGCTGTCTCTCGCCGGATGGCTCTCCAA
GTGCTCATTGGTGCTGCAGCCATTGGCTCCAAGGTTTCACCTGCAGATGCAGCCTACG
GTGAAGCTGCCAATGTATTTGGCAAGCCAAAGACCAACACAGATTTCTTGCCGTACAA
TGGAGAAGGATTCAAGTTATCCATCCCCTCAAAGTGGAACCCAAGCAGAGAGAGGGA
ATTCCCAGGACAGGTTCTTAGATATGAGGACAACTTCGATTCCAACAGCAATGTTAGTG
TCATAATCAACTCAACTGATAAGAAATCCATCACCGATTATGGTTCCCCCGAGGAGTTC
CTCTCCAAGGTGGACTTTTTGCTTGGGAAACAAGCCTACTTTGGCAAAACTTCTTCTG
AGGGTGGTTTTGACAATGATGCTGTAGCAACAGCAAACATATTGGAGGTTTCAAATCC
AGTGATTAATGGAATTCCGTACTACTTCTTGTCAGTGTTGACAAGGACTGCTGATGGGG
ATGAAGGTGGCAAGCACCAACTAATTACAGCAACTGTTAAAAATGGTAAGCTTTACATT
TGCAAGGCACAAGCTGGAGACAAACGGTGGTTTAAGGGAGCAAGAAGGTTTGTGGA
GAGCACTGCTAGTTCTTTCAGTGTTGCCTAA

LOC115995090 (CAMTA):

ATGGCAAAGTCGAAGAATCACACAGCTCACAACCAGTCCTACAAGGCCCACAAGAAT
GGCATCAAGAAACCCCGCAAGCACCGCCACATCTCCACCAAAGGGATGGATCCAAAG
TTTCTGAGGAACCAGAGGTATGCCAGGAAGCACAACAACAAGAGTGCTGAGGGTGCT
AGCGAGGAAGAGTAG
