# Supplementary material for: Transcriptomic Analysis Reveals the Opposite Regulatory Effects of WRKY and CAMTA Transcription Factors on Total Tannin Production in Quercus fabri Fruit
Source: Int J Mol Sci. 2024 Dec 6;25(23):13103. doi: 10.3390/ijms252313103 (PMC11642043; doi:10.3390/ijms252313103)
Supplement: Supplementary file 1 [file ijms-25-13103-s001.zip › Supplementary Document S2.pdf]

Supplementary Document S2. WRKY and CAMTA transcription factor target gene promoter sequences and cis-acting elements

UDP-glycosyltransferase (UGT):

LOC115963067 promoter:

CAAAGAAACTCACAAAAATGCAAAAGCAATTTGATGACATCATTCATCAATGCTGAGT
ATTTAAAAAAAAATAAAAAACAATTTTTCTCACATGGTGAATCGTAAATAATAAAATTAT
GAACTTATTGGCTGGGTTTGGATCCAGCTTTTGCGTTTACGTTTCTTCATTTTTTTTTTT
TTTTTTCCTGCTGCTGACTCGTTTAGGGAACAATGGCTACCGTTCATGTGAACAGTAAC
CGGCAATTGTTGAAAATTGGGTCCTACAATACTATTCACACATTTAAAAATTATTTTGCT
ACAGTATTTTCAGTTTTCAGTTTTCAGTTTCAACAAAATAAATTCTATCCAAACAGACC
CATTATTTTATTTTTACTACTCAGAGGATCTATTATTTTACTTTTACTGCTCAAAGGTCAC
CCAAGTGGCAAATTGGAGCAAAAATTGTGGAATTTTTTTTTTTTTTTTGTCCCTGGCC
TTTTATAATTTATTTCCTTAATACCTACAAAACAAGTCACGTGTGCAAAAGTAAGCTGAT
GAAGTGGTATTTTTTTAATAGGAGTTTTCCTTAGAGGTTGTTCAGAAAATGTCTATACTA
CTTTTATGGAAGATATAAAGAGTTTAAAAAAAATAGTTTTTCTATAAAACTTTATATATAT
ATATATATATATAATAGTATACAGTTAATAGATGATATAAGCATTTGTTAATAGATTAGTTTT
AGAAATGTTTTATGAAAAAAGAAAATACAATTAATTTTTTTGATAGTTTATATATTTCTTG
TGAAAGTAATATTAAAATTTTTCTAAAAATGGTTCATTAATAAATGTCTTAAGAGCACTC
ATTAACCAGACTTATATATATATATATATATAAGCACACAAAATGTTTAAACTTAGGGAAA
ACCATAAAATTATTTAGGTATAGTATCTTAAGTTTTACAATTAAATTCAAATATGTGATTT
TGGGACTTGTAATTAACTACTTATAGTAAGAAAGTGCATTGGTCATATAGTTAATAAAAC
ATAAAATTGTGCTCGTGTAGCACTTAAAAAGCTATAAATAGTTTTTACCCAAATTGTAAT
AACTGATTGAAATCCAATATTCAACACGCTAAAATAAAATCAGGCTGAAATCCAACAAC
GTTAACAATTTAAATTTATATAAGTAATATATATATTCAAGAAGGAAAGTATTTCCAGGCT
ATGATATGTTTATCTCTATAATAATTGGATTTTTTTTTTAAGTGCATGGTTTGATCAAACA
ACGAGATATATATATATATATATATATGTATATATTACTAACTTTTATGACTTCTATTAGAAAT
TCTCGTTTTAACCGTTGAAATTTCTTTGATTTTAGGCCACATGTGCAAAAAAAGGAAAG
TCGAGCAGAGTTTCTTTTATTTTTATTTTTTGGTAAAAAAATAAAATGGGTAGAGGGGT
GATTAGTGTGATTTCTCTATTTAAATGTGACTATAATAACACCTTATTTACTATCAGACAA
GACCCTATACTCTTGATCTATAAAAACTCACTTATTATTTTTTAGACTGTTTAAAATAAAA
ATAAAATATAAAAAGTGCTAACACACAACTAATTATTGAGGAACGAACCTAAATACTGG
GACTTACTGGTATCTAACCTACGAGGCTACCCGAAAGTTGGTAGAATTTCACAAGCTAG
TTCCAATTTCCAAAGTGATCCGTATATAGTTGCTATGACATGGCTTTAGAAAGCTACGCC
ACTCGTGATTGTCAAAATCAACTAATCAAACAGGTCTGCTTACATATTTAATTACATCAT
GCATTCTTTTTATAAGTCTATCACCGATCATTAAATATACATGCAAACCAATATTTCAAAC
CTACCAGTCAACTTTTCTAAAAAAAAAAAAAGATCCTACCAGTCAACTTCGAAGCATC
ATAAATATCTCTCCTTGTTTATTGCTTTTCTAAGTTCTAACAAATACTTTTGTGTGTTCAT
TGAGTGAGAGAAAATG

serine carboxypeptidase (SCPL):

LOC115969198 promoter:

GGGCTCATCCCTGCTACCAATACTCGTTGGAGTGATTCTTTATTGCTGGTAGGATGTATG
TTTGAGCCATATTTGGCACGTCCGAGGAGACATTCCTCCTCGGACTGCCTTTTAAATAA
GTTCGGGCCCATAAGAGTTGGGCCGGGAGCCCTTTTGGCGCCCCACTTTCTCCTCTGA
CGTGGCTGGACTCTGTATGGGGCTCAAGGCCCATTGTTTGATCTGGGGACTTTACCCCT
ACAGTGTGTATTTTTTTAAAAAAATAAATAAGGTAATGTAGAATAATATTTAAAAGTGAC
ATAGAGACCGTGTGCTAATTTTTAGGCAGAATGGAGAAAAAAGAAGAAGGAAAATGC
CTAAAATTTAGGAACAAAAAATACTCTTTTAACCATTTTTTTTTAATTTAAAATTATTTAA
CTAAAAGATAGGGATATTTTAGAGAGTTTAAGAGTTTGTATGAGAATTTTAGTATGCAA
AATGATGAAACCCAAACCGGGAATCTTGTTATTAGTAGTATTAGACTCATAACGCATACT
TTGCACATGCAATAATGCTCTTTTATTTATTTATTTGTTTTTTAAGTTTAGTGTTATAATGT
TTAAAAGTGATATATAAATAACCATTTGTAATTTTTTTTTAAGAAAGAGGTAAGGTAACA
TGGAATAATGTTTAAAAATGAGATATAGATAGTGTCATAAGTTTTAGGCTGAATAGAGA
AGATAAAAGAAAAAGCCTAAAATTTAGGAATAGTAAATTACTCTTTTAACCAATTTGTA
TTTTTTAATTTAAAATTATTTAACTAAAAGATTGGGGAATTTTAGAGAGTTTAAGAATTT
GTATGAGGGTATTTTAGTACGTAAAATGTTAAAATTGAAACAAAAAATCCTGTTATTAA
GATAAGATAAGACGCTCAATATCACTCAAAGATTTTGAACTGACTTTGGTGGGCTTCGT
TTGAACTTCGTTATGTCTTACCTTTGTGAATCTATGGGTTTCTAATGGCAAAAATTGGAT
GATTTTTTTCTAAAAGGATTTTGGACAGAAATCAATATTTCAACTAGAATGACCGAAAC
ATTCTGAAATTGCTGAAATCATCCAAAATAGTCCAGCGAAAAAGTGATTTGCAATGGA
GGGTTATTTGTACTAGTTTGGTCACCGGTTAGAGAAATTTGGGCCTGTTTGGTAGGCCA
TTTCAAACACACTTTTGAGCATTTTAAACACACTTACACATATTTTCACAAACATTTTCA
CCCACAAATATATTCAAAACATTTAAACAACATTACTCAAACCCCTCTACCAAACACCC
CCTTCATAGCCAATACGGTTTGAAACTTACACCCTCTGAAAACAAAAACCACGTCAAA
TTACATTTAAAATCATATAATGTAGGGTTGGTTTCAAAGTAAACTTTATAGTGTCAAAAG
TTTCAAATTAAAGATGTTTAAGATTATTACAAATTAAGCCCTGTAGTTTTTGTTAGTTCA
AATTGAATTATGGAGTTTAAAAAAAGTAGAAGGTTTAAATTCATATGATTTTATAAGTTT
AGGGTTCAAATTTAAAACTAGAGAAATTATTAGGTTTAGTTTGAAACCATATTTAAGTAT
AGGATTTAAAATATAATTTAACACGGGTCCCCACCACCCAAAAAAAAAAAAAAAAAAA
TTATGGTCCAAATGTGCAATGATGCAAAGTACAAAATTCTCTCTTTTTTTAGGTAAAAA
ATTGACTCTACCAGCATAAGACATCCTCAGTGCTCAATTAAACAAATGTACATATTTTAT
TACTACTAAACTCAACTCAACAACATTCCATCCAACGTTCTTGAGCTTTATTTTCTTGAT
TATTCAGCTAACCTTCTATACCTACATTTTAGTATGATAGTATCAAACAACTAACTGCATG
AGCATAGCTTAACTAGTAAGTTTGAAGCCTAGTAATACTAATATTAGTAAATATTTTAGG
GGCTAATTGATAACCAAACCATAATCACCTTTACATTAATG

LOC115969101 promoter:
TAGTTCGAGTGCAATTTCGACCTCTCTCAATCTATTGAAATTTAAAAAATAAAAATAAAA
TAAAATAAAAGTTTTTATTATTTTTTTATTTGAAGTAGAAAGATTTGAATATTCAAAATGA
TATAAAATGAAATATAAGAATTAATTTTGATTTCATTACTCTTTCTGTATTCCATATGAATT
ATTTTTAAAAAGTTGTTTCCATAAAGAAATTTACACTTTAACTCGTGTATTCAATATACTC
CCTAAAAAAATCCTTTAAAAAGGAGGGAATTAAAAAAAAAAAATTAAAAAAAAAAAAGGG
ATCTACATTAAATATAGGTAAAACCATGCAGATATATAGTTTATATATATAAAATATAAGAA

TTTTTTGGAACCTTTGAAATCCGGTATGCACGTTGGTAATAGTACATAATAATAATAAAT
CAATAATAATTATAGGTTATATATATATATATATATGAGATGAGTTCAAGTTACATTTAGTATA
AGCTCTATTAAAATTACACTTTTTTACATCATAGATCTTTAAGAGATTTAACGGTTTAAA
AAACACCATTAAATATTAATTCCTTACCAACTCATTTACCTAATTAATAGCATTTTTTCTC
GCTCCTTATTTATTACTTTCCATATATTTTTTTTATTTTTTAATGGTTGAGATACTATTCTTT
TCCACCTCATTAAATGTCATTCTGCCATTTTTTCAAGAAACTTTTGAAGCCTTAAAGGTC
ACCTCGCCATAGCCAAAACCTCCAATTTTAGCCTTGTAGTACCCTGATTGAAAGGTTTT
CTTATTGACACTATTTCTTAACCCCAAAATAAAAAAATAATAAATAAATAAATATAATAA
AAGAATTGCAAAGATGTCATCTTTAGTAATTTGTGGCTTGATAGGGCATTTAACTATATC
GCGGCTTTTGTTTTAATAGGGTACCAATGAAATTTTCCTAGCAAGGAGATATCAATAGTT
TTTCACTCACTCATAATCGCATTATGAAACAATTTTTTTATTTAGCCTTTTGGCAACAATA
TCTACTAGTATTTATTCATGAACTTGATTGTTTGTAGTGGCGTCAACTATATATAAACTGA
TCTTTCAAATTTTTTTCCTTGGATAGTTTCGACTATTTGCTTGTTCAAAATATTACAAGGT
TGTGTCTGTTTTTTAAAATGTAAAGTGGGAACTTGCTTCTAATTTAATAGAGTAGTTCTA
CCAAAAGAAGAAGGAGAAGAAATGGAAGCTAGGAGGGTAAGAATAATTAAGGAGAG
AAAAAAAAATTGCTATTATTTAGGTAAATGAGTTGGAAATCGATAAATAATCAACTATGT
TTTTAAAACCATTAAATCTTTTAGATATCTACAGTGTAAAAATGGTGTAATTTCTATTAAG
TGTAACAAAATATATATATATATATATATATAAATATATATATAAATAAAGTAGGAAGCAAAT
GAAATGTGTACCACGTGGAACAATATTTAAGTTAGGTTGTACGTACCCCGTTTTATTGGC
GGCTACTGAAAGTTGGTTGTAATTGAATTTCTTCTATGTACATCCATTCATCAATTTTAAT
GGCCTGAAAGTCAAAGCAAAGCATGTAGTACTGTTGACCTTACTCAATTAGTTCACCTA
CGTATTAAACATAAAATTCATGAAGATTTTCATTTTTGAATGGAGTGAAAGTTAGCTGC
AATTTTATAGGTAAGAAACTATTTTCCACTGTTGTTTGATGAATTGACTCCAACTTCAAT
GGAGCAAATGTTAACAAACCTTCATAACTTGGACCTACGGTTTGGGGGAAAAAAAATG
ATTTTTTCGTGGCAAATACGAAAGAGTACCTATCAAACAGATATGATCAACGCCTCTTT
TTAAGTTTCAAAGATATTCACTATTATCTGTTAGAAACAAATGGTATGAGTAATATTCATA
TATCCTTCAATTTTTTTGGGCACATTCTTGCTTTCCGTATCACCCACCACAACTAATTAA
GGCATAGCAAATATG

carboxylesterase (CXE):
LOC115960284 promoter:
AATGTATCCAGCAAGGATAAAAAGTAGCCATGACAAAGTGAAAGTAAGAAATCCATTA
ACATTGATCTTAAGGGGTCCGATTAGGCTAAACTTCAAAATCCAACAAGACTAAACTC
ACGAGATCTAGCAAGATTATATAAATAATTTCAACAAATTTAAATAGAGCAAAGCAAAG
AAAAAGAATCATATTCAACAAAGAATAAGAAAGAGTTTAGAAAACTAAAGTAAACATG
TTGAGCAATGATAGTATAAAATTTTTACCATGGTCAAATAGGAACTATCCAGCAATGTTG
AGAACATAAAAGAGTTGAACAAAGTAATACAAACATCTTCAGATAGATTATATAAACAT
GATCAACATTGATTAAAGAACTAATCAGTTAAATAATTAGTAAGGCAAGGAGAAAAGT
GAGTTAGAGAGGGGGAGGACACAGCTTTGGGTGTTAGATGCCGATAGTAGGTAAGTTG
CTTTCTCATTTCTCTAATTGCTAGAACTATGTTCCATTCTACTAGATTTTGGATGCTGACT
ATGGAGGAAAATCAGTTGAGGCGGGTAGACATACTTAACCCCTAAAACGACAACAAG
GCAAGGGTTCAACATATTCTAATAACAAACTAAAGTTCCAAAATACTACTATGCTTAAA

AATTGAATTTAGCATCTCAATTCCTACTTTACACAAAAGATATCTCTGGTGTAACAAAAT
GACCCAAAATGTGTTTTCTTAGGTTTCTTGATTTTTATCCCAATAATAATCAAGTTGCTG
ACTCTATCTATACATCCCTACATCCAGAAGGAATGGGAATTGTTAGTTGAACCTCAAGC
ATGAGACTCAATATCAGGACCAGCCCAAGATAGTTTGAGGGCTAATGCGAAAATTGTA
AAGGGACCCTTTTTATATCTAAATATCACTTAAATGATATTTTATTTTTTATATTATAATTTT
TTATTTCAAATTTATTATTAATTCTTACTAATTAATAAGACTAATTCATCCAAAGTGAGTT
AATGATACTTATTATGTAGATTTTACAAACTGACGAGTTATCAATAAATAAAAGTTATTC
AAAACATTAATTTATTATATTGTTTGATCAGTTTGTAAGTTTTTTGTCATAAAATTTGTATT
AGTTTTAGCATTTTTCATTCACTTAATGGTGAATTGGTAATTGTGTTGTATTTCTTTTTTT
CTTTTTTTTGTGTGTGAAGAAGATGCTAAAGTACTATAAAAAAAACACACAAACTAATG
TGACATTTCAAGAAAAGAATAAATAAATATTGAATTACTTATTGTGATTGAACATATCAA
TTTATAAGGCCCATGTGATAGAAAAAAAATGTGCAAGTTACACACTCATTTTTTAATTAG
AAAAAAAAAAAAAAAATACTGGCCCCATTTTAATTTGGGGCCTTTCTATGTTAGGGGGCC
TTAGGCCATGACCTATATGGCCTAGACCTAGGGCCGGCCCTGCTCACTATTTACAGAGA
CACCCGGAATTCTTAAGTCCTCTCAGGTCATAATTCTCACTCACCTCGGCTTTCCGAAT
CATATTAAGATCTATGAGGGTTTTAAGGCACCAAGGCAAGGGTGTAGTAGGAGAGGAA
ATAGTACATTTGACTATTTGAGTTTTGGCCTTTGGGATGTGGATTCAATCATGTAGTACT
AGAAAGTAGATACAACATAAAAGTTCGAGATTGAAGCCAAACCTATGATTGGTTATCGG
ACATCCATAAATTAAAGTCTCAACACGACTTCTAGTTGTTACATATACCAAGTCATGTTT
CTTTTTTGTCAAAGTGTGCTGGTACGTTTTAATTTAATGAGGAGACCGAGAATTTTAGT
TGGATAAGACTGTTAGATGAAACTAAACATCGGCAACCAATTATTTCAAGGAATAATTC
TTCCTCTACATAAATTGAATAACCAAGAAGACCTTAATCTCTCACTTTCCTTCTTTGTCA
ACAAAGCTAAACACTCCAAGAATATTAGTTGCTTCTTTCAATG

LOC115956969 promoter:
ACTGTGAGAGAGTGAGAGAGATGTGACTACTTCTTCAAAGTAGATCTTGATTCTTTGG
GGCCGGTCCTCATATGAGTTTTGTGAGCTCTCCCATTTTGGCTTCTGGGTTTTGAAAAA
TTCAAATTTTGAATTAAAATTATATTTCTTTTTGGTTAGGTTGGAACCGTGTACTTGAGT
TTCTCAATATTGAATCTGGGTTTTGTAGAATTGTAAAAAATGTTAAGGTTTTAAGGGACT
GGTTCTTGTATAGTGGACTTTGTGTGCTTTGTTTTAATGGAGTTGTTTGTGTACTCGTGG
TTGAATTCTGTACGCTTGAGAGGTAAACATGGCTCCATTAATGGATTTTTGTAGTTAGCC
AAAAAAAGAGTTAAAAAGAAGGGCAATCCTAGTAACAGTAAGATTCTACCTTTCGGTA
AAAAAAAAAAAAAAAAGGTGTAGTAAATAGTAACCATGTAGAACCACAATTTACTCTA
TATTAGAGGGTGAGTTTTACATTTTAGCCTAATATAAACTACTCTTCTCTCTCTCTCTC
CAATATGGGAATAAAAAATATTGTTTAATTTTTAGCAACTTATTACAGAGAGCATTTAGC
ATTGGGGGTTGTAAACCCCCCCCCCCCCCCAAATTGCAATTTTTACATCCCAAAAACA
TTTATTAAAAAAAAAAAAAACCCACTTTATTCTCTCTCCTCTCTCCTTTATTTATTCTCTCT
CTCTCTCATATTTATTTTATTAGTTAGTTTATATCATTTTATTAGGTTGTATATAAAAATAAG
AACTGAGATGTTAAGTGTATTGTTAAATGCGATAGTAAAATAAATAAAGTAGTGTTTTAG
AGTGTAAAATGTGTATATTTTTACAAAACCAGATGTGAATGCTTTAAAGTTGGTATTGAT
ACCAACTTACTATAGCAAATTGGTATAATTTTTTACATATAGCAACTCGGATGTTGCATGT
TTTTAAGGGTTTGGGTTGTAAAATAGCAATTTGGAAGGGGGGATTTACACCCCAATACT

CTAAGAGCACTAGCATCTTAGGATGCAAAAAGAAAAAAAAAAAAAGAAAATATCCTCA
AACACCACTTTATGTATTTTACTAACTCATTTTACAACTTACCAAACATCTCAGTTCTTAT
TTTTACATACAACCAAATAAAATAATATAAACTACCTAATAAATTAATATAAAATAGGAG
AGAGATGCGATTTTTTTTAAATTTATTTGGGAAGACAGAATAAAATATTATATAATAAGTA
TACAAACTCACAACCTATTTACCAATTTACTGTTCATTGTTGCTAAAAAAATTTAGCAAT
AGCAACCCGGATAAAATTAGTGTTTGGGGCATTCGGATGTAAAATAGCAATTGAAAGAT
TTTTACACTCTCAATGCTAAATATTAGCTTTAAAACATGTGAGAGTAACGACTCAAAGA
AGGCCGCTTATTTATTTATTTATTATTCAAGTATTCACTATTTATATACATTCAACTCTTAA
CAACTTTTGATTCCAAAAAAAAAAAAAAAAAAAAAAAAACTCTTAACAACTTTTGATCT
CAAAAAAATTAAAAAAAAAAAAAAAAATCCCTAGAATAGACTCACACTAAAAACAGTG
TCAAGTATTACCTTTAGATCACACGGCATACCTTTCTCAAAAAAAAAAAAAAAAAAAA
TCACACGGCATACTTTCTTTTGGCTGAATTTCCTGATGAAGTATGCCGTGTGATCTAAAA
GGTAATAACTTGACACTGTTTTAGTGTGAGTCTATTCTAGGGATTTTTTTTTTTTTTTT
TTTTTTTTTTTTTTTTTTTTTACGTGTAAAAACATCAAAACATGATGATTTTCCACTGAG
AAAACTTTTCCATGGAAAATTTTTTCCACAAATTAATTTACTACTCAAATATGTCCGACA
AGTCTCCTCTGTACTGTTGAAGTCGTTAGATAGAGAAATCCGAATTTGTGGTTCATACT
CAAAGAGTAACAACATCTAGCTAAGGTTGCCATG

phenylalanine ammonia-lyase (PAL):

LOC115953247 promoter:

TTTGATTCATTGCTTAAATCCGATCCATATATTGATGTTGATGATGTCGTGGGGTTATGAT
CCCAATTGATGAAGAGGAAATGTGGTCCATGAATCCGCCTCTTGAAGTAAGGATCTTAT
GGGCCATGTGAGCCCATTCCATGAGATATAAGCTTATTTTGGGTTTTAAAGAGATTTACC
CAAAAAATCAATAATATGTTGACGTGGCGTAGGTTGCCAATGAAGTAATGCCATGTGAG
GCGAAAAAAGAGTCTTCAGCATAATTACATAAGTTTATTTAACATGTTAGAGTACATTAC
TTTATCTTTTCTTTCTCTTTATTCCAATGCCTCTGAATTTTCACATAAAAGTGTTAACAGT
TGGTTCTTTTTCTTTATCCAATGCATCTAAATCTTCACATAATACTGTTAACAAACGATGG
AAATGGGAATGGTGATGGCAATGGCAAAAGCATGAACAGTGATGATGGTTGTAACGCC
CTAAAATCATAAGTAATAAAAGTCTATTTAATCTGAATAACCCATAAAAATATTAAATAA
AAGAAACCAGCAATCTTAAATCTCATCACAAATGTCAGAACTCTAATCCACCAAAACT
AAAACGTCCTCAAAATAATTCTCCAATACAATGTTTAATGCCATAAAAATAATAATAAAA
TCCTCAGTTCCACAAAATAGTTCGTAACTGCTGTCTCCCAAGAATCTCTACTCCAATAA
TCCCTCTAATGTATCTGTAATGGGAAATAAAGGGGGGTGAGATAACTCAATAAGTGGAA
TTCACTAACATTGGGGTGTGGGAAAAAACCTTTCAAATAAATAATTCTTACAACAATTT
CATAACTTGTAACTCATTAATATTTATCATCAAATACTTCATATGTATAAAAAAATATCTTT
ATATTGTGAGCCATCATAATATATATTTGTTGGGACTGAGGTTCTACTAGAACCTAACTG
AGAGTTCAATATTTTATTTTCAACCTCCACCAACCATATCAGTTAACCATTTTCATAACCA
TAGTCCGAAATAAATCAAATAATAGACAAAAATTATTGCCTTTTTTTTTTTTTAAATGTCT
CATATATTTTTTAGACAAACACTCATTTTAAATTAAAAAAAGCCTTAAATCTCTACAAAA
AAAAAAATATAGTATGGAATTTTTTTACTCGAGTAATCCTACAGCCACGTACTAATAGAT
TTTAATATTAAGTGTGTATTTGACAATTGATATTATACTTTTTGATACTATTTACAACTTTT
TATATTTTTCAAAAATGTTGTAAAAAAATCTTTTTTTTTTTCTATTAAAATTTTCTAAAATA

TTTTATAAAGGCATCCGTTAACTTTTCTTGACCCAATCAGTTGTTTTTTTTTTTTGAGAA
AGACCCAATCAGTTGTTTTTATTTTTTTGCTAAAAGACCCAATCAGTTGTTTTAATTTGT
CTTGTGCGTGTTTACGGCTTGCGTGCAGCAACTTTTACCCTTCTACTAATAACCCAACC
CGGGCCCCACCAAATAAAGACTAAACATGCATTAGTGATGCCACGTTGCGAGTTCTCA
ACCGTGGGATAGAAATAACAATCCTATGATCAGAATTAAGACTTTCAACCAACCCCCAT
GGGTCCCTCACGGAGCCACCACCTTTGTTGTATTCTTTTCCTCAAAGCTTACCTACCAA
AATACTCTGCCACGCCACTGAATAAGAGTTGTTTGGTGTGGTAATTTAAGTAATATTTTT
TTAATTTTTAACTCACATGTTTCTAAAAAAATACCAACAATGAACCACGTCAACAAACA
GCGCTTAACAATTCAAAAACTCCATGCCAATGCCATGCATGTTGCCATCTATTTAAGCCT
CACAACCCTTCTTCACAAACCAGGAAATCCTTACAAACGGGCCTAAGCTAGTGAGCAA
GATTTCCTATACTTTTTCATTCAAACCCCAAAAACTCAGTTTTTTTCTTGCCATTATTTCT
CTCTTTCAATTGGGTCATTCGTCGACATG

LOC115973406 promoter:
TGGGCTATGAGATTTGATTCATTGCTTAAATCCGATCCATATATTGATGTTGATGATGTCG
TGGGGTTATGATCCCAATTGATGAAGAGGAAATGTGGTCCATGAATCCGCCTCTTGAAG
TAAGGATCTTATGGGCCATGTGAGCCCATTCCATGAGATATAAGCTTATTTTGGGTTTTA
AAGAGATTTACCCAAAAAATCAATAATATGTTGACGTGGCGTAGGTTGCCAATGAAGTA
ATGCCATGTGAGGCGAAAAAAGAGTCTTCAGCATAATTACATAAGTTTATTTAACATGTT
AGAGTACATTACTTTATCTTTTCTTTCTCTTTATTCCAATGCCTCTGAATTTTCACATAAA
AGTGTTAACAGTTGGTTCTTTTTCTTTATCCAATGCATCTAAATCTTCACATAATACTGTT
AACAAACGATGGAAATGGGAATGGTGATGGCAATGGCAAAAGCATGAACAGTGATGA
TGGTTGTAACGCCCTAAAATCATAAGTAATAAAAGTCTATTTAATCTGAATAACCCATAA
AAATATTAAATAAAAGAAACCAGCAATCTTAAATCTCATCACAAATGTCAGAACTCTAA
TCCACCAAAACTAAAACGTCCTCAAAATAATTCTCCAATACAATGTTTAATGCCATAAA
AATAATAATAAAATCCTCAGTTCCACAAAATAGTTCGTAACTGCTGTCTCCCAAGAATC
TCTACTCCAATAATCCCTCTAATGTATCTGTAATGGGAAATAAAGGGGGGTGAGATAACT
CAATAAGTGGAATTCACTAACATTGGGGTGTGGGAAAAAACCTTTCAAATAAATAATTC
TTACAACAATTTCATAACTTGTAACTCATTAATATTTATCATCAAATACTTCATATGTATAA
AAAAATATCTTTATATTGTGAGCCATCATAATATATATTTGTTGGGACTGAGGTTCTACTA
GAACCTAACTGAGAGTTCAATATTTTATTTTCAACCTCCACCAACCATATCAGTTAACCA
TTTTCATAACCATAGTCCGAAATAAATCAAATAATAGACAAAAATTATTGCCTTTTTTTTT
TTTAAATGTCTCATATATTTTTTAGACAAACACTCATTTTAAATTAAAAAAAGCCTTAAA
TCTCTACAAAAAAAAAAATATAGTATGGAATTTTTTTACTCGAGTAATCCTACAGCCAC
GTACTAATAGATTTTAATATTAAGTGTGTATTTGACAATTGATATTATACTTTTTGATACTA
TTTACAACTTTTTATATTTTTCAAAAATGTTGTAAAAAAATCTCTTTTTTTTTCTATTAAA
ATTTTCTAAAATATTTTATAAAGGCATCCGTTAACTTTTCTTGACCCAATCAGTTGTTTTT
TTTTTTTGAGAAAGACCCAATCAGTTGTTTTTATTTTTTTGCTAAAAGACCCAATCAGTT
GTTTTAATTTGTCTTGTGCGTGTTTACGGCTTGCGTGCAGCAACTTTTACCCTTCTACTA
ATAACCCAACCCGGGCCCCACCAAATAAAGACTAAACATGCATTAGTGATGCCACGTT
GCGAGTTCTCAACCGTGGGATAGAAATAACAATCCTATGATCAGAATTAAGACTTTCAA
CCAACCCCCATGGGTCCCTCACGGAGCCACCACCTTTGTTGTATTCTTTTCCTCAAAGC

TTACCTACCAAAATACTCTGCCACGCCACTGAATAAGAGTTGTTTAGTGTGGTAATTTA
AGCAATATTTTTTTAATTTTTAACTCACGTGTTTGTAGAAAATACCAACAATGAACAACG
TCACCAAACCGCGCTTAACAATTCAAAAACTCCATGCCATGTTGCCATCTATTTAAGCC
TCACAACCCTTCTTCACAAACCAAGAAATCCTTACAAACCTGCCTAAGCTAGTGAGCA
AGATCTCCTATACTTTCATTCAAACCCCAAAAACTCAGTTTTTTCTTGCCATTATTTCT
CTCTTTCAATTGGGTCATTCGTCGACATG

chalcone synthase (CHS):

LOC115994574 promoter:

GTTATCTGCAAATTCTTAAGCCTTTACGACACTTCAATTTCAATAATTTTAGTCATCCAA
GTATTGTGTTATCCTCCTAACACCCTAATTAGGGGTCACGCCTTTTATTACCAAAGCCTT
ATACCTCTCATGACATGAAACTATCAGGGACTCACATAACAAGTACTAATACTGACCTG
TTCATCAAAAAAGTGCAAATACTGACCTTCCGATTTTATCTATTTTAATATAAAAATCTAT
TTCTTTTATTTTACCTAACTACTTTTGAAAATACTTACATCAAATTATTTATTATAAACTCT
ATTTTATTAAAAGCCTTTTATTTTTTATAATTATTTTTCTCTCTTTTACACACCACAGCTAC
TATCTATCTCTTCTTTCAACTTCAAAAAATGCAAAGAAAGAATAAAAATTGAAATATATA
ACAATAATGCTTGTGTATATTTGCACAAGTATGATAACAAAATACATTTTGCACAATAAA
TGCATAATAAATCAGATGATTTTTGTGGGTTTGGTTGCAAAATTTGGAACTTATGTTATT
TTACATCCACGAATGCTAGGGATGGCTCTACATTGATGTCAAGGTGGTCACAGGATCGC
CCTGACTTGGGAAAAAATAATTATTATATATAAAAAAAATAAAAATTTTATGATCTTTTA
ACTTTTAAAAAAAAAAAAAAAAAAATGTGATCATCCTAAATTTTTCTAGACCCAATGTAA
ATAAACTTTGGACAAAATTTTGCAACAAACTTGATTGTAGATTAAAGCTACAACTTGAC
TTAATATTTTTTTTATTAAATGTGAATTTTGAAAAATCCACCTTCTTATATCCTTCATTCTT
GCAAAAAATCAATAAAATCAAAGATCAATAGCTATGTCATCAATTAAATATTTAAAATTT
GAGTTTTTTTTAGTCTTAAATTATACATAAAAAGAAATTTATGGATCAAATAGTAAATAA
CATTTGATTGGCATGAAATTTGATCTTTTTAAAAAACATAAAGAATATACAATTTTACTG
TTAGATTTTCAAATTATGTAGTCATATTAATTTGTTTAGTAAGAGTTGTAGCCTTAAGCTA
CAATTACGTTTGTATAGCCAAATTTCTTCTCCAAATAAAAGTTTATAACCAAATTTGATC
CTTAAACTTTAGTCTCCTTAACAATATATTTGGCCTTTACAAACAAAAAAGAAAAGCCA
AAGAAAAATTTATTAAAATTTTTTTTTGAGAAAACTAAAATTTTGAAAAAGATTTAGT
TTGTAACATTGATAATGAGACTATTATGTAACAATTTCAAAATAAAATAAAAAAATCGTA
GAAGGAAATTGCAAAATTTTATACATTTACATGTTTTTGTCAATATATAAAATTCTTTTT
TTTTTTTTATTCAATTTGTATTATTTAAATTTCATAATGACCACCTTGCAAAATTTATAGA
GCCATCACTGTCCAATACATATATATAAAAATAAAAATAAAACCTTCTACCGTGTGACAT
ATTACACAAGTGTCCAAAATTTGGGGTGATAGTTTTGGAGTTGTCATGCATGGGTACAC
AAAATACAAAAATGAGAACAGTCAAGAATTCCAATACAAAGGGTTTGGTTAATAATAG
GGGATGATGATGACGATACAGGATATATAGTTGTTTGTTTTGTTTGGTAAGTGAGACGG
GGAGAGCACCAGACCACCAATAATAAGAAAATCATGCGGTGGGTTTGAAAGGCAAAC
CTACCAAACCCACTTGTCACGTGCCAGCCTGTAGCCTTACTAGTTTGTAGTAGTTGTTG
GAGTTGGGAGCTTCACATAAGGCATTGGTCAAGCACGTGATCCCCAGCTACCCTCCCT
TCCTCTCAAAAATCCTATATATAAAAACACTCCTTCCCAACATATTGTGACACCCACAAC
TTAAAATACAATCGTAGCCACTGCAACTAGCTAGGTAGAGAAGGTGCTAAGCTTTGAG

CTTTTCTTTTAGAGAAAGAAAAAAAAAATG

LOC115976336 promoter:
GCTAATTACCTAGGTTAATCTGATTGTGGTTTTAAGGGGTCTAAAAACTATCAGTCTCCA
TCTTCATTCTTTAACATGAGAAGCTAATGGTCCGTTTGAATTAAGGGGGAGTAGAGTAG
ATTTAGTCTAAAATTAGCCTATTTTCAGCCAACTCTATTCTACTCCCCTCCATCCAAATG
GGCCCTAAGTTTAATTACCTCACCAAACTGGCAATAATAGTAAAAAATACAGGGAATCA
TTAGTTCTAAAGAAACACAGGAACCATTAACAAAAAACATTTGACTAAAGTTCAGCCA
CCGATCTTCCATGTTGGGGGGGAGACTTCTTATTTTTAAACAGACTTGTGAAGAAACAT
TATGGGCAGTGTCTACTCCATACATGCCTGCAACATGCTATTTTTCCCGAGCATTATTTCT
TATATATATCCTCAAGACCCCACCTGAGTATTAGTCAACGAGATTAAAAATTGAACAAG
ACCTTCCTTAACACGAAGCCAAGTTTAATGACCTCACCTCACCAAGGGAATCATTAATT
AATTCTAAAGAAACATAGGGAACCATCAAAAAAAAAAAAATGACATGACTAAAGCTAG
ACCACCTCTTCCAAGTTAAGGAGTCTTCCCATTTTTAAAAGACTTGTGAAGAAACCTTA
TTTCTCATATCCTCAAAACCCTATCTAACTATTTAGTCTACATATACGAGTATAGATATGA
AACAAGACCACCTTCAACATGAAGCCTAGTTTAATTATTCCAATTACCATACCAGCAGT
AGGTAGCTAGGTGAATTGTCTCAGCCCAAACTTTGGGCCGTTATTATCCGTCTCTTCTCT
TCCTCAGAATGTCTATTAAGATGTTCTCGTATTATGACAATATGACCATACGAGAGAGAT
GGTCTGGGACCATAATTAACTGCCTTGCCTTCCTGTGTTATATTTTCCTTAAATGCAATG
TCTGTCCGAGGTGAAGAAAAAAATGGTAGATCAGTAGGTGGAGTGTCCATACTTGTGA
ATGGTACACCATCTAGTCAAACATTAATACAAGGCATTTAGGCCTTCTTTAGGACAAAAT
ACTTTTTCCGCCAATCACCTCCAAGTTTCCTCTCTTTTTTCTTTTTTTTCCCTTCTTTTTT
TTGTTATAAATCTTGTTTCTGTCTCTTTTTTTTTTTTTAATATCAATTTTTATAATTTCAAA
AAATTTATTTAGTCCTAGGAAACAAAACATACATTGAGTGAGTTAAATTAGGATCACAA
TAAAAATTCATAAGCTAATATTTACATAGTACTTCACATTTGGGTCCATCATAATTTACAA
TTTAGAATTTGTATATCAATGGTTCTTTTATTTTTTTTATTTTTTGATAAATATAATAGATTT
TCAACTAAATGCATCCGTTCCATGATTATTACTCTACATTATTGGGCCAAGAAATTAATTA
GTTTTATGATATAGGTCAAGTCTAAACCTTAAAAACTCTTATTCAACGACAAATTTTTTA
TCAATTGAGTTAACTAAAACTTATATTTTGAGCTATTCATTCGTGAGTATCAAGAAAAAT
AAAGGAATATTGTTAAATTGTTCCGAATTGTTGTTGGTGAGGTTAGTTAAAAACATAAG
TGGCTTATAAGAGAATATTGGAAACAGAAAGGAATAGAGAAAAGATCTCAAAATGTTA
GGAGTCAACATGCAAACCAACTTCATGAATAAATGTGGCTCCATATCTACCACCGGTTA
AAGTGCCTAACCCCAAAGCTCACGTGGATCACCAAATGGCCGTCCATTCAACCTACCT
CATTCCGTCTTATATAAACTCTGCCTTTGCTCTTCTTGTCATCTCGTCCCCAAAAACCAA
AACACAAACAAACTCTTTCAGAAAATTAATTTCAGTATCACAAGCATCACTTAGAGCTT
AAAAAAGAAATTTTAGTCCGATCGAGTTCTTGGTTAACTCGGTCTTTGCTAGCTCTTTG
AGAGAGACTAAGATCAAATTCAGGCTGTCACACATG

leucoanthocyanidin reductase (LAR):

LOC115991055 promoter:

TCACTGCATTGCTAGGCACGGTTTGCCCAAAACCCTCATCACACCTCACCTTAAATTGA
TTAGTCACTATGCAGTTTTATGTTGTTTAGTCAATGTCAGGTTGGGCGTTTTATTCTTATT
TTGGGTTGGTTTGAGCCAAACCGGATATCTTTTTTTTTCTCTTTGTTTTAAGCTGATTA
AGAGTAGCCCAACAAGTTTTTAGTTGTGAAAATGTCTACAGTTGTGAAAATTTTAGTAA
TTTTTAATATAAATTTTAGTTATATATTTAATATAAATTAAATAAATAAAATATAATAAATAT
ATAGTATGAGGAGCAGTTCGGTTCGGTTTTTAAAAAACAGAATCGCATTTGCCCTAATT
CTCGCCTAAGTAGAGTCAACCTTTTGTTTCTAAAACTACATAGTATGACATATGTGGGGA
TAAAAGAACTGAGTAGAACTATTGGGCCGTGGGTCATGCCTGAGGATGCCAAGGAACC
CGAGGATAATCAGACAATAGGATAAGCATGTGGGTTAATGGGCCAAGGACGAGGACAA
ATAGCGACGAAAAGGTGGTGCCTCCCGAGGATCCGAACCTCCTCAGTATGCGTTGTGG
AGGGGTGGAGTCTAGCCGCCTAGAGAATACTATGCAAGAGGTAACCATGTTTTTAAGG
ATAAGGTTTAGAGAAAGGAGTCAATAGTAAACTAAGAAATATCTGAAAAGAAGGCTGC
TACCACCACATTAAATATTCTACACCTAATCCCCTGGCCGCATTTATGTGGAAATGGCGC
CAGAATAGGGATATTTAGTCTTACAACTACATTCAAAGCTTCTAGGAGGGTTCTGATTA
GACAAGCATACAAGAGATCATCTACATGATTAACAAGTGGAAGGTTAGGATAAAGGTA
GAGGGGAAGACTATATAAGGGGAGGGCCTCCATGAAAGAGGGGGCATCTGGTTTGCA
GAAAAGAAAGAAAGAACTCTTTGTACTTAGAAAAAGTTGGAATAAAGATAAGAACTTA
TCTTCCTCGGATTTGACCGAGGAGTGTTTTTCTTTTCAAATTATACTTCTTAATCATTCTA
ACACTAAACCAATCTACTGTGGCCCACACTTGATTCATTAAGTCTTCATCTATAAACTCC
ACTCTCTAACAAATTTATTGTTATGGGCTTGTTGGGCCATCACTCACTTCTTTTGGGCTG
AGAGTCAATTTAGGCCTTCCAACATAAATTTCTCGATTAAATCAACCATTTTTTTTTATAT
AAAAAACTTATAACTTTTTTAAGAAGAAGATAAATTCATTATATCATGAGAAATAACGG
ACTCAATCAACGATGAATTTTTCTCTATTTAAGTTACATAATTATCCACCATTTTAAATATA
TGATAGACTCCTCTAAGAAATATTGGTATGCGTGTAGCTGAGGTGCTCTACCAACTAATT
TAGAACCTCCTGTTCTTGTTATACATATATTTAATTATGTTGATACTTTGTTGGAATTAATG
ATCCCTAATCAACAATAGAATATATATATATATATAATTTTTTTTTTTTTTTTAAGAAAGGATT
TGTATTATCCCTAACGAATCAACCGAATTAAAGAGATGAACTCGCACGAGGCTCTTAGC
TATCAGTCAAAGGCATCAAAAATTGGTTTTAGCTAAACTTCAACTAGTGGATTTTTACC
ACAGCACAAACAAGAAGGCTATGGTGAATATTGTCCTGTTTTTGTGGAAGAGTAGCTG
AAAACATAGTACCCTTAGCATCCCGATAATATTTATTATCAGCTTTCTCTCATATGTTTTTA
TCTAAGGTGCTCAGCCCCACTAAACTTCCGATTATATATAGGTAGACTTGAATACATGCA
CTTCATTGTACATAGCAGCCGTGCCAACCTTGGCTCTCCCAAACCTGTGTCTCTGTAGA
AACCTCGGCCTTTGTAAAACAAAACTAAGGATCAAAACTAGAACTTGGCCTTTGTGTA
AAAGAAACCAAAGGACAAACTCATCACTGCCAAGCATG

LOC115963778 promoter:
GTTAAGTGTTTCACTCCAAGTATAAAAGGAAAAACCCTAACTACATTTTAGTGGTCTTT
GTTGAGTTGTGTGTTGAGTCTTTTGTGAGATCTGAGAGGTTTGTACCTTCAAACACACG
CATAGAGCTATCAAGATCAAGATTCACATCAAGAACTTGATGGTTGGTTTAGTTACTGC
ATAAAGAACATTCATGGAACAACAAAACCTTTGAGTGGAGTCTCAAAGTCACAAGAA
GGAGAACTTGTGGTTGCTGCAGATTCAAGAAAGAAATAGTCCATGAATTTGGAGCTTG
CACGTGGTCGTGCCAGTAAGTCCTATGTGTAGTAGCATTAGGATGTTAGTGATCTAAGT

CCTTTTGTAAACTTCAATTCTTTCTATAGTGGATTTTTTTTTTACCTTGAGAATAGCTATA
TTAAATCCTCCCTAGGTTTTTTACCAGTTTGATTTTCCTGGGTCATCAAATCTTTGTGTTC
TTTACTTTCTGCATTATTTCTGTTTTGCTCATAATTGTTTAACCTAGACTTGAATAACAAA
CTTGTTAATCAACTTGGCTTAATATTAGGTTAAACATATTGTGTTAAGGGGTCTAAAAAC
CTAACAAGTGGTATCAGAACAGGTTAAATCTTGTGTTAGATCTCTTGATCTAAGAGTTG
ATTTTTGACCCCTGTTGTCATGGAACACAGTCACTCTCTTGTGATCCCTCCACATTTTGA
TGGGAATAACTATGCCTACTGGAAGGTTAAGATGAAAGCCTTCTTGAAATCTATAGATG
AGAGAGTTTGGAACTCTGTTGAATACGGATAGGAGAAACCGGCTACTCCTGTTAGTGA
GCGGTCAACGTCCCAAAAAGAAGCAGCCGCTTTTAATAGCAAAGTTTTGAATGCTATT
TTTAATGTTGTTTCTATGGAAGAGTTTAAGAGAATCTCAAATGTTGAGGTTGCTCATACT
GCTTGAAAATTCTCCAAATTGTGTATAAATGCACAAAGGCAGTTACCTAATTTTTTATAA
TAGTCCCACCTCCACCTACTTAAATTGGATTAACTAGTATGCAAGTATCCACCTGTACTT
AAATTGACTCATCCTTGACTAAGAACTTGCCCCTCGATCGGTTAAAAACCAATTAAATA
CTGCAGTTTATGGTAAATAAGTATTGATCTAAAATTCGTTGGCTATTTAAGAAAGACAAT
GTTTGTCAAACGCGATTCAATTTTAAAATTATATTTTCAACTCACTATTTCAGTTCAAAA
AATTATGTATGTAATAGTGCGCTTGTGTAACAAGCAAAATGAGGCGCCTACATATATGTG
TTGCACTCATATAATGGGTGTGATTGAAAAAGATGTAAAATTTACATTTTTTTTTTTAAC
CTAAAAATGATCCAATTCAATGAGTATATAATTGTGTAAAATTAATTTTATAAATAATTTG
ATGTGGAGTGTTTTGAAAAAAAAAATAAAGAAAAAAAAATAATGCTAATTAAAATAAA
CGCACACAAAAATTACTATCTAACAGGTTTTATCCATTTTGGAGAATAATAAAATTAAAT
TTAGAGAATTAAAAGTGAGCAAGTACATGAATCATATTAATATTATAAGACAATAATCCT
TTGGATCAATAGAGTACTTCTCTTATACTCAACTACCAGTCGAAGATGTGATATATCAAT
TATCAAACCACTTCCATTTTCTTTTTTTAAAGTACATTTTTTTGGGTAAGTAATCACACCT
CCATCAAACATTCCACTCGAAAAAAAAAGACAAAATATAGATGAGACACCGAACAAA
GTTAGCCAAACCAAAGCCCACCAACCAAACCTCACTCGCTATGCGCGTGAACGTTACC
AAACATGCATAAGGTCCACTCTTAGTCTTATACCCACTTTTATATAACCCCTCACAGCCC
CTTCCCTCCTATCATAACTATTGTGTGCCTTTTCTTCTTCTTCTCTACAGTTTTTTGCTTTT
TTATCATTTGATCAAAGAAACTGAAGAAGAAAAAAAATG

anthocyanidin reductase (ANR):

LOC115957898 promoter:

TTTTTTTTTTTTTTAAGAAACCAATATATATATATATATATATTAATAGGCAAAGTTTAGAA
AAAAAAAACCCAATTAAATTTTAATTAGATTTTTAATTTTGTTCTAAGTGAATTATTGAG
TGTAAAAATTGAAGAGTCTAATCTAATTAGATTCTAAATTGAATTTTAATTGATCCAAAT
TTTCCACCATGTGTTTTTTTAAGTTTTTAATTTTTGTGGTAAGGAAATTATTGTGTGCATA
AATCAAATAGTCTAAAATCGATTAAATTCTAAATCATATATATATAATGAAAACCATTTCA
TATTTTAAAAATATATTATTTTAAAAAAATGTAAACTTATTTGAATTGTATATTTATGATTA
TTTTTAATTGTATTTGTGTAAGGGTTACATGCTACTTTATTGATAATAGTAGACAATTGAT
GTATTTAAATGATAATCTAATAATAATAATAAAAAATAGCAAACCCAAGGCGTATCACCT
ATACTATTTAAATAATAATTTTTAATATTTAAATAGTATTACATGTATTTTATATATGTATTTT
TAAAAATAAAAAACAACGTTATTAGAATAACAATACTAAATGAGTCCAGGTGGCCAGT
GGTGTACTTGTATCCATTGATTTTGTTTTTTATTTTCTTTATTTTGTATTGGCCGTTTTTTA

TTTTCCAAACAAAATTTGACTTTTATCTTGAATCCTTGATACACCTGAAACAACTTTGGT
GAGGTCGGTAGTTAAACCAGATGAAATAGCTACATTTTAGTGTTATACATACATACATAC
ATACATACATATATATATATATATATATATATATATATTATTGTGTGTATATTTATACATGCAATAT
AATAAACTACTTAATTTACTGATATCATGAATTATTTTGTTTCTGAAAGTATCAAATACAA
TTATATAAGATGACATATATATGCTACTTAATTGAAAAATATTTAAGGAATATTTAATCTAA
CAAATATAGTATTTTTTAAATAAAATCTATAATATATATATATATATTTCTATTTCTATTTTTCCC
TATATTTTATGCTAAAATTTTTATATATTTTTCAAATTTTAAAGTAAATAAAATATGTATATG
TTACAGAAAGTATAAATTCCATGTTCTATATTACTACTAATTAGCTCAATTGGATCTTTTG
ACGGCGAGATTTCCGAAAAAAAAAAAATTGAAAAAGGAAAAAAGAAATCTCAACGCTT
GACAGGGTGGCATACGTGTTGATGTCACGGCCTCACATGCATGCATGTGACCGTCACA
CAACAAAACTTGATTTATCTTTACACAATTAAGAAATTAAGGTTTCATACATGTGTTTAA
AAATTGAAAATTATTATTTAAAGATATGTATGAAAATATGTGTGGACAAAAAAATATGTG
AATATATGTATAATGTTATTTAAAAGCTGAAAATTGTTGTTTGAAAACACAATCCAAACA
CCCCCATAAAGACCAAACCCATTAAAATGCAGATACATATCTAAAACTAAACACATTTC
TGTGTCCCTTTTATTTTACAGCCACCAAAAAATCTTCTGTCCCCAATGTAATATCTTTGC
CGTCAGATGAATATGTACAAATGGCAAAGATAATTTAACATGTAAAAGGCATGCATTGA
TGACATAGACCATCATAATCTCCAAATTTTTTAATGTTTATCCTATGATGTAATTGCATTA
CCATCTTAGTCAGCCATATCACATTTAATATAAAATGTACCATTATGACCCGCAAATATGC
TTAATTCTTTTTCTTTTTTTTAATATGACCCGGATATATGCTACACCTGTGTAGTTGTTCTT
AATTTAATACAGTAATTTATAAATATAAAATATAAACAATCTTATGCAGTTATGAATGATG
GGAACTTTCACCATCATTAATGTTATATAATTCCCTCATCATAGACAGTTGACCAGAATG
AAAGATTTATAAGAGAGGTTTCTACTGGAGTGAGATGGTTTAATCTTTAAAGGAAATG

LOC115992511 promoter:
CAATAAGAAGTTGGTTATATCAACATTTTGTAAAAACATTGTAAAATAATTTGACTGTAG
CATTTCTCTAAAAAAAGTGGTGTTAGTGATGGGTATGTCTGAAAGTATGTTACATCAATC
ACATTTTATCATTTCTACAAATTATAAAATAAGTTATGAAAAAAGTTATGGTCCTAATATT
ATTCTAATTTTTATTTTTATTTATTTTTTTCTAGAATTTGGGTGAGCTTGTACCTTCTAGCA
TGGTAACAAAAGTTTTGTGTTTCTTGAACTTATTTTCCTAGGTAAAGCTGGTGAAAGGA
CTAATAACCTAACTATAATGCCTAAAAATTGAGTAGGCTTTCAATTCTTGGTGTTTTATG
TTGTGAGAATTTGTTGTGACAATAGAGTTTTTTATACCAGTTTATTATGATAGCAAAAGG
ACAAAAGCTCTAATACCAGTTTGTTCTAATGGAAGCTCATGTCCATGAATAAATCAACA
CAATATCCAAATAGAACAAACCCAAATGCCTATACCCTTACTTTATGTAAAAGAAAAAA
AAATGTAACTATAGTATTATAGCTTAAGAACACCTTGTTTCGTACTGATTTTCAAAATCA
CGTGTTATAATTATAATTAAATGATTAAATTTATTTTTTTTAACATTTTAAACTTTAGAGAT
GAAGTGGTAATTTACCATGGTATATAATATACCACATGTTTGATTTTATATATTTTAGATAA
GAAAGTGGTATCTCAACCTTTAGTTATATTACTCAAGTCTTTATTTACGAGACATTTCTA
AAATTCAATGGACCTATAGTGTGGTAAATTAAACATGGAACCTTTAGGTTTATGTAATGC
CCCATACCAATGATCCCATCACCAAATTACCCCTATAGAAGTTATCTATTTTAAACTTTCA
TAATTTTATAAGGAAAGAAAATAAAATATGTAGCAAACTTAAATTTTTTTTTTTAGAGTG
TTTTAACCTATGACGTTCGCTCCTGATAATAATTATTTATCATCAGACCAAGATACCAATA
AGTTTTTACTGATGGAGTTGATGAGCTTTCCGTAATGGTTTAGAGCTTCCTCAACATCCT

CCTTTTATTGTTATTATTTTTTGTTCTTTACGGGTTTTTTCCCCTTTTTAATGCGATAAAAT
GGGAGATATTGGTTCAACCCAGAGGAAAACAGAAAAGGGATCGAGAACAAGAAATCT
TCATTTTGAAGGAACACTACAGGGACCATCTTTTGTACTTGCTGGTTAATTAAATAGATA
AAGTCATAAAACCTCCTCTCTTTTCACCAAAAAAAGAAAAAGAAAAAGAAATATTGGA
CCTATGAAAGTAAAATAATCTTTTTAATTTACGTTAACACCCTTGATGAAAATTCACATA
ATTACTCAATTTGTACACGTCTTTTGTCTGTGGAGGGTGCTTAAGGGTTTTTTAAATTCC
TATTTGGTCCACATTATAAAATAAAAAAATAAAAAAAAACCCTAATGTAAGATTCTTCA
AATCAAAGATATGAAAACATGGGATTGGGAATTAGACAATTCATATATATAAAAAAAAA
AAAAGAAAAGAAAAGAAAAAAGATTGCAATTAATGTGGATGGTAATGTCTAAAATAAT
GTGCAATGCTTTATCTTGTCTACCCATCTCAAGTTGTTTGGTGAAGTCAATGGTATTTTG
ATTATTCTAGTGAGTGACATAGACAATACAACAGTGATAATTTAAGAATCCTCTAGACTC
CTTAAAGATGGTTTTCAACGTAGTTGGTGGCGCACAAGCAACATCACGTGCTCACCTT
CTTCTTTTTCTTCTTCTTCTTCATCTTCTTTCTCAATTTCGAAATCAACGAAAGTAACTC
CGTTTAAAAAAGCCAAACCCCACAACTCAAATTTAAAGACTATATATATGTATAACTAA
CACCAAATTTATTTTCTCATACACAAAACTAAGTATCCAAAGTAGTGTTACCAAAATCA
AAGAAAGTAGTTTTGAAATTCGATAATG

The yellow highlighted sequence is the potential binding element of WRKY, and the green highlighted sequence is the potential binding element of CAMTA. The red ATG is the start codon of the gene.
