# Supplementary material for: Transcriptomic Analysis Reveals the Opposite Regulatory Effects of WRKY and CAMTA Transcription Factors on Total Tannin Production in Quercus fabri Fruit
Source: Int J Mol Sci. 2024 Dec 6;25(23):13103. doi: 10.3390/ijms252313103 (PMC11642043; doi:10.3390/ijms252313103)
Supplement: Supplementary file 1 [file ijms-25-13103-s001.zip › Table S3. Primers used in this study.pdf]

Table S3. Primers used in this study.

Primer Name	Primer Sequence	Description
P-*LOC115963067*	**F:** CAAAGAAACTCACAAAAATGCA<br>**R:** TTTCTCTCACTCAATGAACACAC	promoter amplification
P-*LOC115969198*	**F:** GGGCTCATCCCTGCTACC<br>**R:** TAATGTAAAGGTGATTATGGTTTG	promoter amplification
P-*LOC115969101*	**F:** TAGTTCGAGTGCAATTTCGAC<br>**R:** ATTTGCTATGCCTTAATTAGTTG	promoter amplification
P-*LOC115960284*	**F:** AATGTATCCAGCAAGGATAAAAA<br>**R:** TGAAAGAAGCAACTAATATTCTTG	promoter amplification
P-*LOC115956969*	**F:** ACTGTGAGAGAGTGAGAGAGATGT<br>**R:** GGCAACCTTAGCTAGATGTTGT	promoter amplification
P-*LOC115953247*	**F:** TTTGATTCATTGCTTAAATCC<br>**R:** GTCGACGAATGACCCAATT	promoter amplification
P-*LOC115973406*	**F:** TGGGCTATGAGATTTGATTCA<br>**R:** GTCGACGAATGACCCAATT	promoter amplification
P-*LOC115994574*	**F:** GTTATCTGCAAATTCTTAAGCCT<br>**R:** TTTTTTTTTCTTTCTCTAAAAGAAA	promoter amplification
P-*LOC115976336*	**F:** GCTAATTACCTAGGTTAATCTGATT<br>**R:** GTGTGACAGCCTGAATTTGA	promoter amplification
P-*LOC115991055*	**F:** TCACTGCATTGCTAGGCA<br>**R:** GCTTGGCAGTGATGAGTTT	promoter amplification
P-*LOC115963778*	**F:** GTTAAGTGTTTCACTCCAAGTATAA<br>**R:** TTTTTTTCTTCTTCAGTTTCTTT	promoter amplification
P-*LOC115957898*	**F:** TTTTTTTTTTTTTTAAGAAACCA<br>**R:** TTCCTTTAAAGATTAAACCATCTC	promoter amplification
P-*LOC115992511*	**F:** CAATAAGAAGTTGGTTATATCAACA<br>**R:** TATCGAATTTCAAAACTACTTTCTT	promoter amplification
Q-*LOC115955609*	**F:** TCCCATTATCCACTTCTCTCACA<br>**R:** TCCACTTAATCTTACAAAACTCCTG	qRT-PCR amplification
Q-*LOC115995090*	**F:** ATATAAAAGCTGCCAATCCTCTCAT<br>**R:** TTCTTGTGGGCCTTGTAGGACT	qRT-PCR amplification
Q-*LOC115963067*	**F:** ATTGAGTGAGAGAAAATGGAGAGAG<br>**R:** GGACTTGGGGGTGAGTAGGG	qRT-PCR amplification
Q-*LOC115969198*	**F:** TGTATTTGCTGCTTCTACTGCTTG<br>**R:** CCTTCCGACTCGACGAAATAG	qRT-PCR amplification
Q-*LOC115969101*	**F:** CCTTCAATTTTTTTGGGCACA<br>**R:** TGTTGCAATGCTTGAGAGAAGTAA	qRT-PCR amplification
Q-*LOC115960284*	**F:** GGCCTCAAGTGACCCAGAAA<br>**R:** GCGGGTTCGGATGAGATTAC	qRT-PCR amplification
Q-*LOC115956969*	**F:** TTTTGATGATGGCACAGTGGAT<br>**R:** CATCTGTGTTCTTCTCTGGAATGTA	qRT-PCR amplification
Q-*LOC115953247*	**F:** TATACTTTTTCATTCAAACCCCAA<br>**R:** CAACTCAGCGAGTCATGGCTA	qRT-PCR amplification

Q-*LOC115973406*	**F:** GCAGTGATGAGGGTGGTCTTT<br>**R:** GCAGCCACCTGTGATATCGTC	qRT-PCR amplification
Q-*LOC115994574*	**F:** GAAAAAAAAAATGGTGACTGTTGA<br>**R:** GTCTTGTGCTCGCTATTGGTGA	qRT-PCR amplification
Q-*LOC115976336*	**F:** CAAAAACCAAAACACAAACAAACT<br>**R:** CATGTGTGACAGCCTGAATTTG	qRT-PCR amplification
Q-*LOC115991055*	**F:** GTGCTCAGCCCCACTAAACTTC<br>**R:** AGGCCAAGTTCTAGTTTTGATCC	qRT-PCR amplification
Q-*LOC115963778*	**F:** CTGAAGAAGAAAAAAAATGTGTGGT<br>**R:** AGGTTGGTTTAGACTGAACAAGAAG	qRT-PCR amplification
Q-*LOC115957898*	**F:** CTACAAGGTTCATGCCACTATCC<br>**R:** CACACCATCACAGCCTTTTACAG	qRT-PCR amplification
Q-*LOC115992511*	**F:** AGTTTTGAAATTCGATAATGGCC<br>**R:** ATCTTTCTCTTATTTTCAGGGTCTC	qRT-PCR amplification
